# Purple Urine Bag Syndrome: A Rare Spot Diagnosis

**DOI:** 10.1155/2017/9131872

**Published:** 2017-11-29

**Authors:** Dilraj S. Kalsi, Joel Ward, Regent Lee, Ashok Handa

**Affiliations:** Nuffield Department of Surgical Sciences, University of Oxford, Oxford, UK

## Abstract

Purple urine bag syndrome (PUBS) is a complication of urinary tract infections (UTIs) where catheter bags and tubing turn purple. It is alarming for patients, families, and clinicians; however, it is in itself a benign phenomenon. PUBS is the result of UTIs with specific bacteria that produce sulphatases and phosphatases which lead tryptophan metabolism to produce indigo (blue) and indirubin (red) pigments, a mixture of which becomes purple. Risk factors include female gender, immobility, constipation, chronic catheterisation, and renal disease. Management involves reassurance, antibiotics, and regular changing of catheters, although there are debates regarding how aggressively to treat and no official guidelines. Prognosis is good, but PUBS is associated with high morbidity and mortality due to the backgrounds of patients. Here, we review the literature available on PUBS, present a summary of case studies from the last five years, and propose the Oxford Urine Chart as a tool to aid such diagnoses.

## 1. Introduction

Purple urine bag syndrome (PUBS) is a rare phenomenon which can be highly concerning and distressing for patients and their relatives. It is a complication of urinary tract infections (UTIs) in which patients produce purple urine in their catheter tubing and bags. This is a simple spot diagnosis; however, a lack of physician awareness can result in misdiagnosis and inappropriate treatments.

Surprisingly, PUBS has been known of for a long time. King George III had blue urine during a bout of chronic constipation [1]; however, the first formal report was in 1978 [2]. PUBS is uncommon, with estimates ranging between 8.3% and 42.1% prevalence among patients with long-term indwelling catheters [2]. Recognition of this entity is important as treatment is simple and can minimise patient and familial distress as well as overmanagement. This article provides a review of the literature regarding PUBS and proposes a method to prevent misdiagnosis.

## 2. Existing Literature

The PubMed database was searched for all case reports concerning PUBS. Studies were limited to those published in English since January 2010. The following keywords were used: purple urine bag syndrome, PUBS, and case report. The reference lists of retrieved articles were also screened. The case reports are summarized in Table 1.

## 3. Aetiology

**Figure 1:**
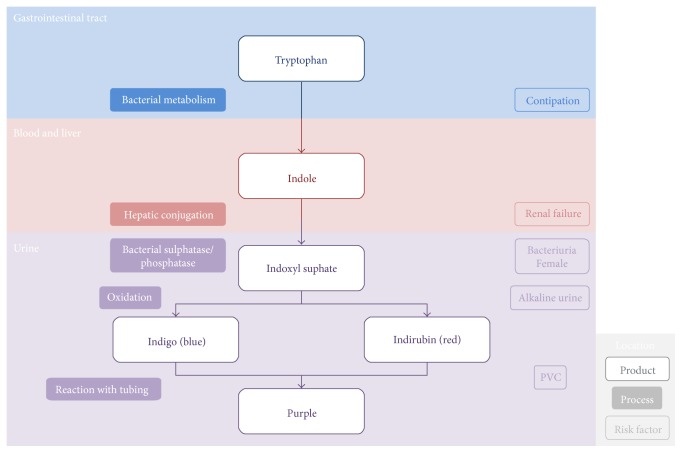
The aetiology of purple urine bag syndrome [5].

PUBS is a consequence of UTIs with bacteria which metabolise products of tryptophan to produce red and blue pigments. This is summarised in Figure 1. Normal bacterial flora deaminates tryptophan in the gastrointestinal tract to produce indole. Indole is rapidly transported by the portal circulation and is conjugated to produce indoxyl sulphate by the liver. This is secreted into urine where sulphatases and phosphatases produced by certain bacteria convert it to indoxyl. In alkaline urine especially, indoxyl is oxidised to indigo (a blue pigment) and indirubin (a red pigment). These pigments mix and react with catheter tubing to produce a striking purple hue. This interaction between the bag (i.e., the plastic) and pigments as well as a high bacterial load is important in precipitating PUBS [3].

There are several bacteria, mostly Gram negative, which have been associated with PUBS. These include *Providencia stuartii* and *Providencia rettgeri*, *Klebsiella pneumoniae*, *Proteus mirabilis*, *Escherichia coli*, Enterococcus species, *Morganella morganii*, *Pseudomonas aeruginosa*, Citrobacter species, and group B Streptococci, though it is often a mixture which leads to PUBS. It is important to note that not all bacteria can cause PUBS, even among the same species, and this is why PUBS is so rare [4].

## 4. Risk Factors

There are several risk factors associated with PUBS. The main factors, summarised in Table 2 and Figure 1, are female gender, increased dietary tryptophan, alkaline urine, constipation, chronic catheterisation, high urinary bacterial load, renal failure, and the use of a polyvinylchloride (PVC) plastic catheter [4]. Female urinary anatomy unfortunately predisposes them to UTIs. If patients have an increased intake of tryptophan in their diet, then there is an increase in the substrate for the PUBS-causing bacteria to metabolise and produce red and blue pigments. Alkalinised urine facilitates the oxidation of indoxyl sulphate to indigo and indirubin, the blue and red pigments which mix to produce the purple colour [6]. Although alkaline urine appears a key factor in PUBS, it is not always necessary, as evidenced by a case report of PUBS in acidic urine. Severe constipation often leads to urinary retention which leaves bacteria in the urine with more time to work on their substrate (indoxyl sulphate) to produce more red and blue pigments [7]. Gastrointestinal conditions such as obstruction, intussusception, and ileal diversions can also increase PUBS, presumably because the bacteria are allowed more time to grow and deaminate tryptophan as in constipated patients. Elderly and bedridden patients with multiple comorbidities more often require long-term indwelling catheters which increase their risk of UTIs; such patients are more likely to be infected by the rarer bacteria which can go on to cause PUBS. Dehydration increases the serum concentration of indigo and indirubin, hence purple urine is more likely. A greater urinary bacterial load during a UTI will obviously increase the availability of bacterial sulphatases and phosphatases which convert indoxyl sulphate to indigo and indirubin [7]. Lastly, renal failure increases the risk of PUBS because there is impaired clearance of indoxyl sulphate meaning the urinary bacteria have more substrate to produce the red and blue pigments and therefore purple urine [4].

## 5. The Risk of Misdiagnosis

While PUBS can be made as a spot diagnosis, a clinician unaware of this phenomenon may misdiagnose it. There are several causes of altered colouration of urine, including haematuria, haemoglobinuria, myoglobinuria, nephrolithiasis, UTIs, food dyes, drugs, poisons, porphyria, and allkaptonuria. Every one of these conditions has significantly different causes and treatments to PUBS, and so, there is a risk of inappropriate management of patients or worse, administration of drugs with several side effects [8].

It is important to understand the wide variety of diagnoses that can be made from different colours of urine as this underlies any potential misdiagnosis. Anything from transparent- or straw-coloured urine to amber urine can indicate that patients are well hydrated or dehydrated, respectively. Foam in or fizzing of urine can indicate proteinuria which may be due to renal disease or excessive protein intake. If a patient's urine is orange, there are a number of possible causes, such as dehydration, UTI, liver disease, biliary disease, food dye, isoniazid, sulfasalazine, and riboflavin. Red urine is assumed to be haematuria but can have a multitude of causes. Serious causes include UTI, pyelonephritis, nephrolithiasis, menstruation, malignancy, BPH, trauma, renal disease, catheterisation, iatrogenic, ibuprofen, rifampicin, warfarin, haemolytic anaemia, sickle cell, thalassaemia, TTP, ITP, transfusion reaction, porphyria, and haemoglobinuria; however, it can be caused by things as harmless as beets, carrots, and blackberries. Brown urine may indicate severe dehydration, paracetamol overdose, metronidazole, nitrofurantoin, haemolytic anaemia, porphyria, or melanoma. There are a number of causes for even darker, that is, black urine: iron, laxatives (cascara/senna), rhabdomyolysis, alpha-methyldopa, cresol, L-dopa, metronidazole, nitrofurantoin, methocarbamol, sorbitol, alcaptonuria, porphyria, and metastatic melanoma. Urine can even go blue-green due to pseudomonas UTIs, methylene blue, food dye, amitriptyline, breath mints, propofol, metoclopramide, promethazine, cimetidine, flupirtine, indomethacin, methocarbamol, tetrahydronaphthalene, zaleplon, biliverdin, blue diaper syndrome, herbicide, and again porphyria. White urine is caused by proteinuria, pyuria from UTI, chyluria, filariasis, lymphatic fistula, schistosomiasis, lipiduria, propofol infusion, urinary TB, hypercalciuria, hyperoxaluria, phosphaturia, lead, and mercury [9]. Interestingly, there is no other cause for purple urine other than purple urine bag syndrome.

**Figure 2:**
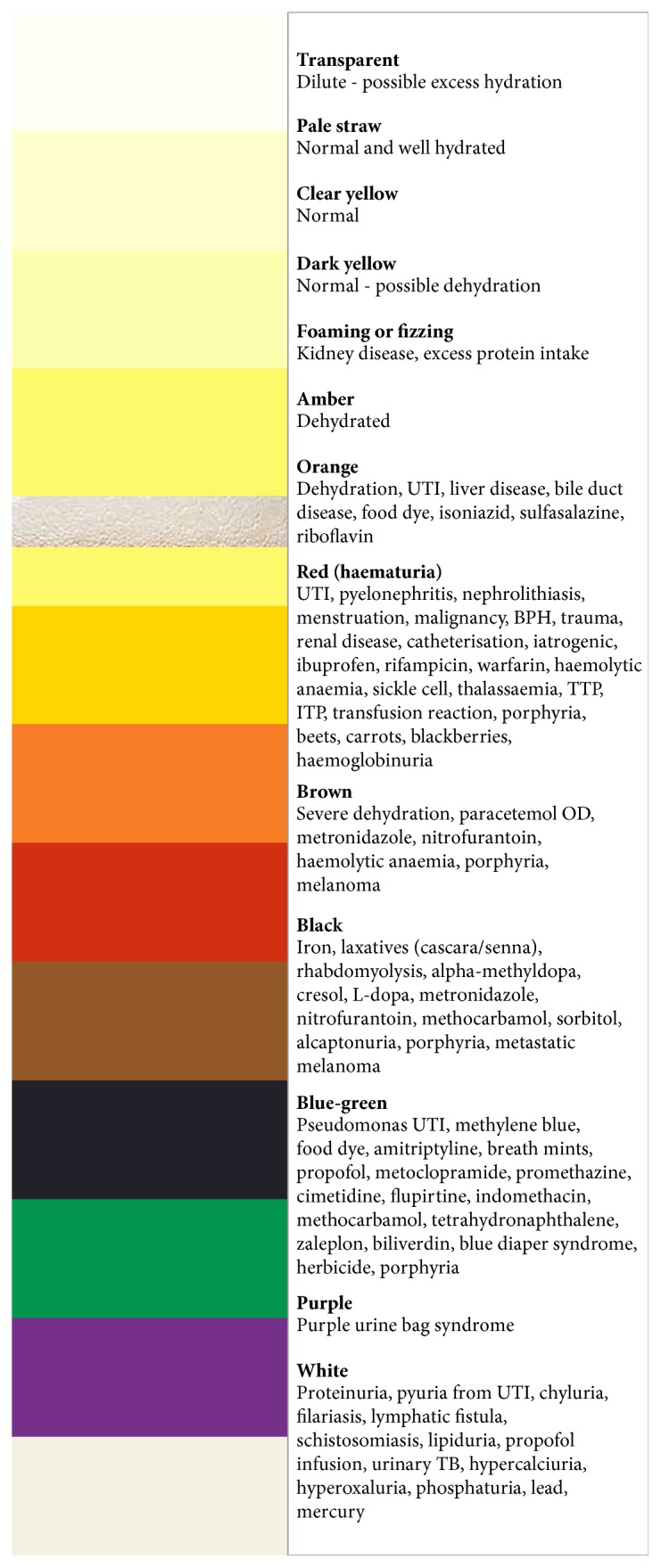
Oxford Urine Chart.

Given the variety of urinary colours and related causes, there is a risk of misdiagnosis; therefore, we have developed the “Oxford Urine Chart” (Figure 2). We believe this would be a useful and quick reference tool for any healthcare staff encountering unusual colours of urine in order to ascertain potential causes. This would be especially useful for nursing staff who have the most contact with patients.

## 6. Investigations

Given the wide range of differentials above, it is important to confirm PUBS diagnoses. There are several factors to consider in patient history and examination. The time course of the change in urine colour is important, especially if it occurs on exposure to air. Infections or tumours are suggested by urgency, frequency, and dysuria, though if an infection is the cause, it may be concurrent with PUBS. Colicky abdominal pains suggest renal stones. A smell of ammonia in urine points to an infection. Certain foods in patients' diets such as blackberries, beets, and carrots may cause a urinary colour change, so it is important to quantify their intake and its time course. Prescription medications including warfarin, L-dopa, and ibuprofen as well as diagnostic dyes might be the causes, and so drug history is extremely important. Pelvic and rectal examination may be required. In terms of investigations, one should start with a urine dipstick test and if there are concerns, then consider urine microscopy, culture, and sensitivity tests and urea and electrolyte blood tests [8].

## 7. Management

It is important to manage PUBS appropriately as it has a high morbidity and mortality relative to UTIs alone due to its contributing factors. One must treat the UTI (e.g., with ciprofloxacin) and any constipation as well as sanitation measures including replacing the catheter. Another approach is to use intravenous antibiotics if the PUBS persists or the patient is in an immunocompromised state [8]. In either approach, it is important to change drainage bags and indwelling long-term catheters on a regular basis to prevent recurrence and because persistent PUBS can lead to Fournier's gangrene which requires surgical debridement [4]. Nonplastic catheter bags are another alternative. Provision of information to the patient, relatives, and clinical team caring for the patient about the nature of the condition and its usual clinical course is essential. There are no guidelines on how exactly to manage PUBS, and these are required in asymptomatic cases that might otherwise progress. In general, it is important to manage PUBS patients on a case-by-case basis [10]. This is particularly important for palliative patients who may be distressed by antibiotics.

## 8. Exploring Cases with Knowledge of PUBS

Agapakis et al. describe an 83-year-old female patient who presented with haematuria and fever on a background of hypothyroidism, Alzheimer's, and colon cancer. She was bedridden and had a long-term indwelling catheter. As a female with colon cancer and a long-term catheter, she was at increased risk of PUBS. Initially, her discoloured urine was misdiagnosed as haematuria, emphasising the need for a tool to prevent misdiagnosis such as the Oxford Urine Chart. Later, urine dip and cultures demonstrated alkaline urine infected with *E. coli*. She was then correctly treated with antibiotics and her catheter changed. Agapakis et al. note that retention of urine discolouration despite a change of urinary catheter may indicate the need for immediate or continued antibiotic treatment to prevent infective complications (such as urosepsis), especially for patients with multiple comorbidities. Until the purple hue of urine has normalised, it is best to continue the treatment [8].

Duff presents a case of a 57-year-old female who suffered with diffuse abdominal pain with a past medical history of transverse myelitis, recurrent UTIs, and two C-sections. She had a long-term indwelling catheter due to her transverse myelitis affecting her urinary control and had a colostomy having needed a bowel resection for bowel obstructions due to adhesions from her previous C-sections. Her gender, catheter, and colostomy all contributed to her risk of PUBS. Urinary investigations found alkaline urine with *Klebsiella pneumoniae* which was treated effectively with ciprofloxacin and changing of her catheter [11].

Bhattarai et al. came across an 87-year-old female patient with altered mental status. Her vast past medical history included dementia, hypertension, hyperlipidaemia, recurrent UTIs, left nephrostomy tube, right ureteral stent, and end-stage renal disease. Her complex urological anatomy, recurrent UTIs, and poor renal function put her at risk of PUBS, not to mention she was bedridden and developed severe constipation. She was found to have alkaline urine with Enterococci and *Pseudomonas aeruginosa* and was subsequently managed with vancomycin and cefepime though changing of her catheter was not specifically mention [10].

Mohamad and Chong encountered a 78-year-old female who was feverish and vomiting with a background of hyperlipidaemia, hypertension, and dementia. Her primary risk factors for PUBS were that she was bedbound and chronically catheterised. Interestingly in this case, urine dip was unremarkable but blood cultures grew *Proteus mirabilis*. The patient was suffering with urosepsis coinciding with PUBS. She was treated initially with ceftriaxone and later with ciprofloxacin. Again, it remains unclear if her catheter was changed [12].

Yaqub et al. describe an 83-year-old female with nausea, vomiting, reduced oral intake, constipation, and purple urine. She had a history of dementia, being bedridden, chronic catheterisation, and recurrent UTIs. Her risk factors include her gender, catheter, recurrent UTIs, and constipation. She had alkaline urine with *Escherichia coli* treated with cefixime, lactulose, and a change of catheter [13].

Al Montasir and Al Mustaque met an 86-year-old female with abdominal pain and a history of osteoporosis, fractured hip, and neurogenic bladder. She was bedridden, chronically catheterised, and constipated. Risk factors for PUBS in this case include neurogenic bladder, catheterisation, immobility, and constipation. This patient also had alkaline urine with *Escherichia coli*. Her treatment was cefuroxime and later ceftriaxone and gentamicin, as well as glycerol suppositories and a change of catheter [5].

Bocrie et al. cared for an 87-year-old female with postfall syndrome and urinary retention for which she had a catheter and faecaloma, the latter two being particular risks for PUBS. Investigations yielded a diagnosis of asymptomatic bacteriuria due to *Escherichia coli* which was treated with her catheter being changed [14].

Keenan and Thompson, unlike in the other cases, dealt with a male patient with PUBS. He presented with purple urine and constipation on a background of benign prostatic hyperplasia and subsequent urinary retention. The constipation and urinary retention for which he had a long-term catheter put this gentleman at risk of PUBS. His urinary cultures grew *Klebsiella pneumoniae* which the team treated with ciprofloxacin [15].

Su et al. were faced with an 81-year-old woman with fever and discoloured urine who was bedridden and chronically catheterised. Her catheter specimen of urine grew *Proteus mirabilis* for which Su et al. prescribed IV antibiotics and changed her catheter [3].

Siu and Watanabe, like Keenan and Thompson, encountered a male with PUBS. This 48-year-old man presented with ischaemic encephalopathy after cardiac arrest. He was a type 2 diabetic and had a previous coronary artery bypass. He suffered from recurrent UTIs subsequent to the encephalopathy requiring chronic catheterisation, increasing his risk of PUBS. He was found to have alkaline urine with *Escherichia coli*. Siu and Watanabe treated this by changing his catheter and giving antibiotics including trimethoprim and sulfamethoxazole [16].

These cases illustrate the risk factors for PUBS very well. They emphasise female gender, alkaline urine, constipation, and long-term catheterisation as key factors in increasing the risk of PUBS. In most cases, the UTI was due to *E. coli* but many other bacterial infections have been found to cause PUBS. A wide variety of antibiotics have been used to treat PUBS in these cases, but oddly, the patients' catheters were not always changed. This highlights the need to increase awareness of PUBS and causation of discoloured urine in general, as this can be highly distressing for patients and their families to see.

## 9. Conclusion

PUBS is concerning for patients, families, and clinicians. It is a complication of mixed growth UTIs whereby the causal bacteria metabolise tryptophan to produce pigments that turn catheter bags purple. It tends to occur in elderly, immobile females with long-term indwelling catheters who might be constipated or in renal failure. This is a spot diagnosis but can be confirmed by history, examination, and urinary investigations. Management involves regular catheter changes and sanitation and possible treatment of UTIs and constipation depending on patient circumstances.

## Figures and Tables

**Table 1:** A summary of case reports of PUBS in the last 5 years.

Case	Key details
Su et al. [3]	81-year-old woman; PC = fever, discoloured urine; RFs = bedridden, chronic catheterisation; Ix = urine with *Proteus mirabilis*; Rx = IV antibiotics, catheter changed
Al Montasir and Al Mustaque [5]	86-year-old female; PC = abdominal pain; PMH = osteoporosis, fractured hip, neurogenic bladder; RFs = bedridden, chronic catheterisation, constipation; Ix = alkaline urine with *Escherichia coli*; Rx = cefuroxime then ceftriaxone/gentamicin, glycerol preparation, catheter changed
Agapakis et al. [8]	83-year-old female; PC = haematuria, fever; PMH = hypothyroidism, Alzheimer's, colon cancer; RFs = bedridden, chronic catheterisation; Ix = misdiagnosed as haematuria, alkaline urine with *E. coli*; Rx = antibiotics, catheter changed
Duff [11]	57-year-old female; PC = diffuse abdominal pain; PMH = transverse myelitis, recurrent UTIs, 2 C-sections; RFs = chronic catheterisation, colostomy bag; Ix = alkaline urine with *Klebsiella pneumoniae*; Rx = ciprofloxacin, catheter changed
Bhattarai et al. [10]	87-year-old female; PC = altered mental status; PMH = dementia, hypertension, hyperlipidaemia, recurrent UTIs, left nephrostomy tube, right ureteral stent, end-stage renal disease; RFs = bedridden, kidney disease, constipated; Ix alkaline urine with Enterococci/*Pseudomonas aeruginosa*; Rx = vancomycin/cefepime
Mohamad and Chong [12]	78-year-old female; PC = fever, vomiting; PMH = hyperlipidaemia, hypertension, dementia; RFs = bedridden, chronic catherisation; Ix = urine dip unremarkable, blood cultured *Proteus mirabilis* (sepsis); Rx ceftriaxone then ciprofloxacin
Yaqub et al. [13]	83-year-old female; PC = nausea, vomiting, reduced oral intake, constipation, purple urine; PMH = dementia; RFs = bedridden, chronic catheterisation, recurrent UTIs; Ix = alkaline urine with *Escherichia coli*; Rx = cefixime, lactulose, catheter changed
Bocrie et al. [14]	87-year-old female; PC = postfall syndrome, urinary retention, faecaloma; RFs = catheter; Ix = asymptomatic bacteriuria (*Escherichia coli*); Rx = catheter changed
Keenan and Thomas [15]	97-year-old man; PC = purple urine, constipation; PMH = prostate hyperplasia, urinary retention; RFs = chronic catheterisation; Ix = urine with *Klebsiella pneumoniae*; Rx = ciprofloxacin
Siu and Watanabe [16]	48-year-old man; PC = ischaemic encephalopathy after cardiac arrest; PMH = type 2 diabetes, coronary artery disease/bypass; RFs = recurrent UTIs, chronic catheterisation; Ix = alkaline urine with *Escherichia coli*; Rx = catheter changed, trimethoprim/sulfamethoxazole

**Table 2:** Risk factors for purple urine bag syndrome [4].

Risk factor	Mechanism
Female gender	Anatomy predisposed to UTIs
Increased dietary tryptophan	Increased substrate for conversion
Increased urine alkalinity	Facilitates indoxyl oxidation
Severe constipation	Increased time and substrate for bacteria
Chronic catheterisation	Increased UTI risk
High urinary bacterial load	Bacterial sulphatase/phosphatase availability
Renal failure	Impaired indoxyl sulphate clearance
